# Complete mitochondrial genome of *Chironomus flaviplumus* (Diptera: Chironomidae) collected in Korea

**DOI:** 10.1080/23802359.2021.1970634

**Published:** 2021-09-06

**Authors:** Kiyun Park, Won-Seok Kim, Jae-Won Park, Ihn-Sil Kwak

**Affiliations:** a Fisheries Science Institute, Chonnam National University, Yeosu, Republic of Korea; b Department of Ocean Integrated Science, Chonnam National University, Yeosu, Republic of Korea

**Keywords:** Mitochondrial genome, Chironomidae

## Abstract

The complete mitochondrial genome of *Chironomus flaviplumus* was sequenced. The circular mitochondrial genome is 15,739 bp and consists of 13 protein-coding, two ribosomal RNAs, and 22 transfer RNA genes (GenBank accession no. MW770891). Results of phylogenetic analysis indicate that the species clustered with other species of the family Chironomidae. This study is helpful to the identification of *C. flaviplumus* larvae, which is difficult to be identified by morphology.

Non-biting midges (Chironomidae, Diptera) are a diverse population containing more than 10,000 species and are found in nearly all types of inland waters (Ekrem and Willassen 2004). Chironomids have been used as indicators for water quality of aquatic environment such as lakes and streams. *Chironomus flaviplumus* Meigen is frequently observed as a dominant species in the highly polluted sites (Tang et al. 2010). Relative abundance of *C. flaviplumus* could be a useful key in detecting diverse level of organic pollution in urban streams (Kawai et al. 1989; Kwak et al. 2002). The discrimination among Chironomidae is generally based on morphological characters of larvae (Makarevich et al. 2000). Cytochrome c oxidase subunit I (COI) gene sequences of mitochondrial DNA (mtDNA) were used to provide a phylogeny of the Chironomidae (Sari et al. 2015). However, there are only very limited number of completed mtDNA sequences in the Chironomidae (Kim et al. 2016; Park et al. 2020). Furthermore, complete mitochondrial genome of *C. flaviplumus* was not available. In the study, we determined the first complete mitochondrial genome of *C. flaviplumus*, which assembled using next-generation sequencing. Specimens of *C. flaviplumus* were sampled from the Yeondeung stream, Yeosu, South Korea (N 34°45′26.0″, E 127°42′51.2″) on May 2020. A DNeasy blood & tissue kit (Qiagen, Valencia, CA) was used for genomic DNA extraction from *C. flaviplumus* larvae. It was stored at Specimen Museum of Fisheries Science Institute, Chonnam National University (accession number CNUISI-020005203). Library preparation and DNA sequencing (100 bp mate pairs with different insert sizes, Illumina HiSeq4000) were carried out in Macrogen Inc. (Seoul, South Korea). The SPAdes (v3.15.2) was used for the *de novo* assembly. The annotated mitochondrial genome sequence of *C. flaviplumus* is available at the National Center for Biotechnology Information (NCBI) database (GenBank accession number MW770891).

The complete sequence of the mtDNA of *C. flaviplumus* is 15,739 bp and was comprised of 13 protein-coding genes, two rRNAs, and 22 tRNAs. Nucleotide distribution is as the following: A (39.5%), C (13.1%), G (9.0%), and T (38.4%). Maximum-likelihood analysis was performed for the protein coding sequences of *C. flaviplumus* mitochondrial genome with other 11 mitochondrial genomes of closely related dipterans using MEGA-X (Figure 1). As a result, the phylogenetic tree shows that *C. flaviplumus* formed a clade with *Chironomus tepperi*, *Polypedilum vanderplanki*, and *Stictochironomus akizukii* in the Chironomidae.

**Figure 1.**
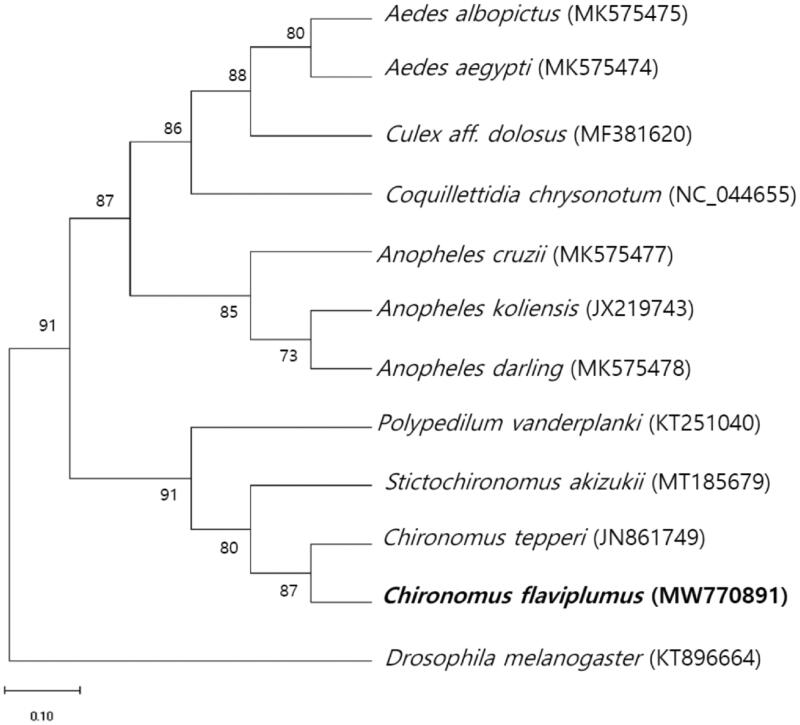
Maximum-likelihood tree is based on 12 Dipteran mitochondrial genomes. All the bootstrap values after 1000 iteration are indicated at the nodes.

## Data Availability

The data that support the findings of this study are openly available in the US National Center for Biotechnology Information (NCBI database) at https://www.ncbi.nlm.nih.gov/, reference number: MW770891.
